# Influence of Surface Energy and Phase Composition on Electroadhesive Interactions

**DOI:** 10.3390/polym17202739

**Published:** 2025-10-13

**Authors:** Konstantin I. Sharov, Valentina Yu. Stepanenko, Ramil R. Khasbiullin, Vladimir V. Matveev, Uliana V. Nikulova, Aleksey V. Shapagin

**Affiliations:** Frumkin Institute of Physical Chemistry, Electrochemistry Russian Academy of Sciences (IPCE RAS), 119071 Moscow, Russia; deathknight_91@mail.ru (K.I.S.); 4niko7@list.ru (V.Y.S.); khasbiullin@techno-poisk.ru (R.R.K.); ulianan@rambler.ru (U.V.N.)

**Keywords:** electroadhesion, EVA, LDPE, surface energy, polar groups, crystallinity, electric field

## Abstract

The aim of the study is to investigate the influence of the physicochemical characteristics of the molecular and supramolecular structure of polymers on electroadhesive interactions and their change under the action of a constant electric field. Currently, this effect is modeled in electroadhesion studies, but the range of variable parameters is limited and includes permittivity, moisture content, and surface roughness. It is important to consider other physicochemical parameters, such as material crystallinity and surface characteristics, changes in which can affect the magnitude of electroadhesive forces. In this study, the electric field strength was varied by altering the constant voltage in the range of 3–8 kV. Polyethylene, ethylene-vinyl acetate copolymers, and polyvinyl acetate were used as substrates for adhesive systems. The influence of the concentration of vinyl acetate groups, which determine the energy characteristics of the surface, and the degree of crystallinity on electroadhesive interactions under conditions of an external constant electric field and without it was traced. The degree of crystallinity was varied both by the cooling rate and the orientation during drawing. It was shown that by changing the polar component of the surface energy and the proportion of the crystalline phase in the substrate, electroadhesive interactions can be increased by 4 times to 120 Pa compared to polyethylene. The obtained laws are explained by the local dipoles induced by polar functional groups, which enhance the polymer’s surface interactions with other materials and external fields. At the same time, the fixation of macromolecules in crystalline regions complicates polarization under the influence of an electric field.

## 1. Introduction

Electroadhesive interactions in polymer-polymer systems, along with mechanisms of sorption, diffusion, and mechanical adhesion, have received insufficient attention, preventing their widespread use in solving applied problems. The use of the complex dynamic effect of electrostatic attraction between two charged objects is due to the large number of dependent parameters [1].

The demand for the use of the electroadhesion effect in various devices has grown significantly over the past few years, particularly in the field of touchscreens to reproduce tactile sensations when operating the device [2,3,4]. In addition to tactile technologies, electroadhesion is also in demand in other industries. Various manipulators and grippers, suitable for working with any material (conductors, semiconductors, and dielectrics) and surface (smooth, rough, clean, or contaminated), operate based on this phenomenon. Such grippers and manipulators have proven to be extremely popular in the production of microelectromechanical systems, since they do not exert a mechanical compressive effect on the fragile object, thereby eliminating its damage. The combination of delicate manipulations of the object and the ability to operate stably in vacuum conditions make manipulators and grippers based on electroadhesion indispensable in the production of micro- and nanosystems technology. Another good example of the use of electroadhesion is the robotics industry. Currently, there are a large number of experimental crawling and climbing robots that operate based on electroadhesion [5,6,7]. These robots are capable of carrying a payload, freely moving along any surface, including vertical walls. The simplicity of the electroadhesion system, consisting of a high-voltage power source and electroadhesive, eliminates the need for heavy pumps and motors required for other adhesive systems, which is an undeniable advantage. Electroadhesion can also find application in the space industry. There are experimental prototypes of a space debris collection device based on the electroadhesion effect, as well as prototypes of astronaut gloves and shoes with built-in electroadhesives to enable movement in outer space along the ship’s skin. The interest in electroadhesive systems is due to their extremely low power consumption in the range from micro to milliwatts, which is associated with the passage of small currents from micro to milliamperes through the electroadhesive at a voltage of the order of kilovolts.

However, despite all their advantages, electroadhesive systems have one significant drawback: relatively low adhesive strength compared to other adhesive systems. In this regard, studying the influence of various physicochemical factors on enhancing electroadhesive interactions in polymer-polymer systems is relevant.

Currently, in addition to a detailed consideration of the application of electroadhesion in tactile technologies, in particular, in touch displays [2,3,4,8], a combination of two adhesion mechanisms has been described in sufficient detail: dry adhesion achieved due to the surface microrelief and electrostatic adhesion (electroadhesion) [5,9,10,11,12,13,14,15,16,17,18]. There are a number of published works devoted to the direct control of the electroadhesive effect [19,20,21,22] and the use of various materials for electroadhesives [11,23,24,25]. Some works describe the production of electroadhesives based on 3D printing, which significantly simplifies and reduces the cost of their production technology [26]. Despite a significant number of works devoted to various aspects of the electroadhesive effect, fundamental studies of the influence of the surface characteristics of materials on electroadhesive forces are extremely limited. There are also few works devoted to the study of the influence of phase composition [27,28] on electroadhesion.

Previously, the authors have published a work [29] that focused on searching for the main dependencies of the electroadhesion effect on various polymer plates and studying the influence of the polymer nature on electroadhesive forces. Electroadhesion in polymer materials (dielectrics) is based on the phenomenon of polarization [30]. Charges inside dielectrics are localized and cannot move freely in the volume of the material, but under the influence of an external electric field they can be polarized, and in this regard, the magnitude of electroadhesive forces in dielectrics is determined by the electric field strength multiplied by the total polarization, which consists of electron, ionic, orientational (dipole), spontaneous, and interfacial polarizations. Depending on the direct or alternating electric voltage, the dominant terms of the equation change, making the main contribution to polarization and to the force of electroadhesion. Thus, when using a high-voltage alternating voltage source, the main contribution to polarization and the force of electroadhesion comes from electronic polarization. However, depending on the frequency of the applied field, the orientational polarization may also become the determining polarization. Whereas, when using constant voltage, the electroadhesive forces are always determined by the orientational polarization [30].

There is a basic approach to calculating the expected electroadhesion force based on the Maxwell stress tensor and having the following form:

(1) $$T_{ij}=\varepsilon_{0}\left(E_{i}E_{j}-\frac{1}{2}\delta_{ij}E^{2}\right)$$

where $\varepsilon_{0}$ is the vacuum permittivity, E is the electric field strength, δ is the Kronecker delta. However, this approach does not take into account the influence of surface energy and its change under the action of an electric field.

This work is devoted to the study of the influence of surface characteristics of substrates on electroadhesive interactions and their changes under the action of a constant electric field. The work also studies the influence of the degree of crystallinity of materials on the forces of electroadhesion. The obtained laws can subsequently serve as the basis for studies of structure formation in multicomponent reactive systems.

The main difference in this work is the investigation of the influence of a number of physicochemical parameters on electroadhesive interactions, not considered in previously published studies. The literature lacks experimental data regarding the influence of surface energy characteristics and phase composition (degree of crystallinity) on electroadhesive strength in polymer-polymer systems. Currently, in the field of electroadhesion, some authors devote considerable attention to modeling this effect; however, the range of parameters used in the models is very limited and primarily concerns permittivity, moisture content, and surface roughness. In this work, the range of physicochemical parameters was expanded, the change in the values of which can affect the magnitude of electroadhesive forces and should be taken into account when modeling electroadhesive systems.

## 2. Materials and Methods

### 2.1. Electroadhesive Systems

**Figure 1:**
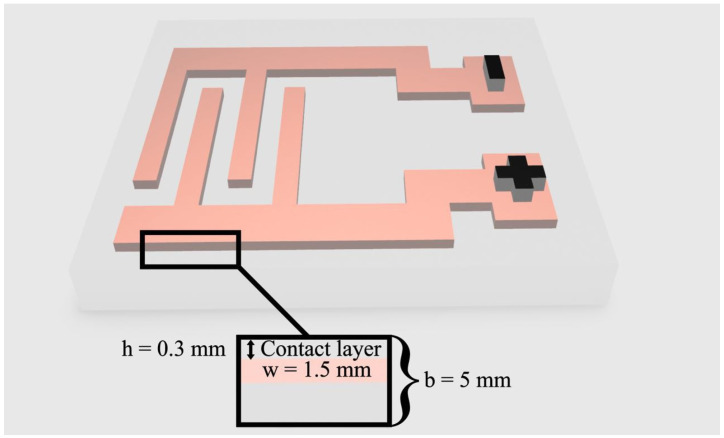
Schematic representation of the electroadhesive: h—contact layer thickness, w—electrode thickness, b—total thickness.

The studied objects were electroadhesive pairs consisting of substrates (polymer films) with different indices of the polar component of surface energy (the thickness of the films was 150–230 μm) and an electroadhesive, which is schematically shown in Figure 1. The complete electroadhesive system consisted of a high-voltage DC source, an electroadhesive pair (electroadhesive and polymer film) and a control system.

The initial materials for producing polymer films characterized by different polar surface energy components were low-density polyethylene (LDPE) (Naftan, Novopolotsk, Belarus), ethylene-vinyl acetate copolymers (EVA) with varying vinyl acetate contents (7, 20, 28, and 40 wt.%)—EVA7 (Sevilen, Moscow, Russia), EVA20 (Total Fina Elf S.A., Paris, France), EVA28 (ExxonMobil Chemical, Irving, TX, USA), EVA40 (DuPont de Nemours, Wilmington, DE, USA), and polyvinyl acetate—PVAc (Sigma-Aldrich, St. Louis, MO, USA). The film samples were obtained by pressing. The degree of EVA crystallinity was varied by the cooling mode and the degree of film drawing. The pressing and annealing modes of the polymer films are given in Table 1.

The electroadhesive was an interdigital electrode with a thickness (w) of 1.5 mm inside a dielectric (Figure 1). Thermosetting polyurethane (NOACAST 700, Composit-stroy, Moscow, Russia) was used as a dielectric. The thickness of the contact layer (h) was 300 μm. The total thickness of the electroadhesive (b) was 5 mm.

### 2.2. Methods

The method of differential scanning calorimetry (DSC) on a NETZSCH DSC 204F1 Phoenix (Netzsch-Geratebau GmbH, Selb, Germany) was used to study the temperatures of phase and physical transitions, as well as to determine the degree of crystallinity of polymer film materials obtained at different cooling rates and after orientation processes. DSC thermograms were obtained at a speed of 10 K/min.

**Figure 2:**
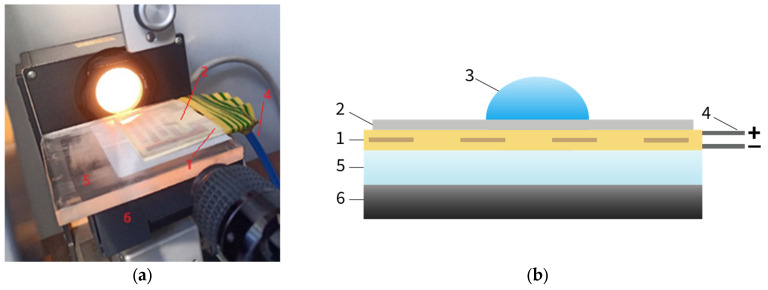
Stand for determining free surface energy by the sessile drop method (**a**) and experimental scheme (**b**): 1—electroadhesive; 2—substrate (polymer film); 3—drop of test liquid; 4—leads of electroadhesive; 5—insulating plate made of organic glass; 6—microscope stage.

**Figure 3:**
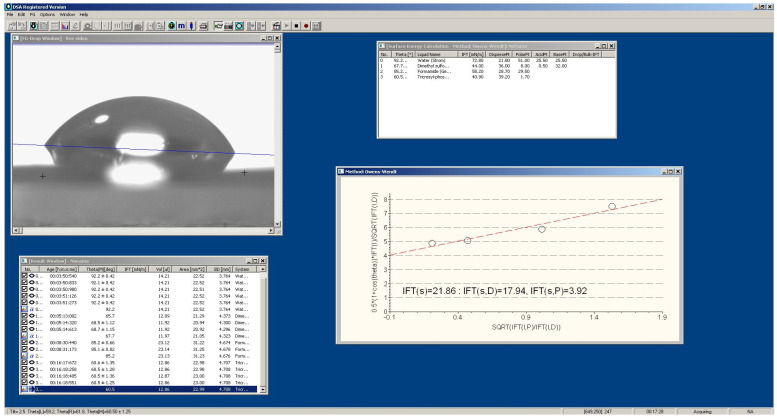
Screenshot of the DSA1 software application when determining the surface energy using the Owens-Wendt method using the example of annealed EVA28 film.

The sessile drop method using the Owens-Wendt equation (Figure 2 and Figure 3) on the FM40 EasyDrop device (KRŰSS GmbH, Selb, Germany) was used to determine the free surface energy of the initial samples, as well as samples under the action of an electric field and after its removal. The following test liquids were used: water, dimethyl sulfoxide, formamide, tricresyl phosphate.

According to the scheme shown in Figure 2b, the electroadhesive (1) was glued with double-sided tape to an insulating plate made of organic glass (5). The polymer film (2) was placed on the electroadhesive (1) and electric voltage was applied to the electroadhesive (1) through conductors (4). A drop of test liquid (3) was applied to the polymer film (2) and the contact angle of wetting for four test liquids was recorded using a horizontal microscope equipped with an angle measurement scale. The free surface energy and its components were calculated using the Owens-Wendt equation in the DSA1 software application (v. 1.29.1.1) (Figure 3).

**Figure 4:**
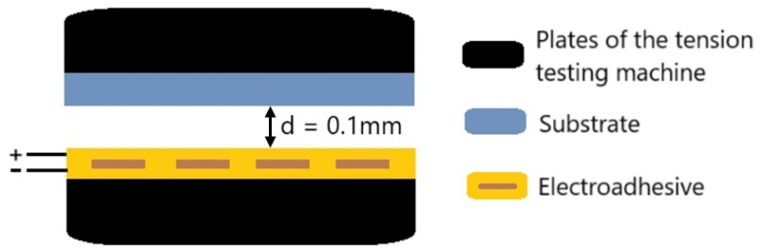
Scheme of the contactless method for measuring electroadhesion forces.

The study of normal electroadhesive force using a contactless method [29] (Figure 4) and the change in the degree of crystallinity of materials using the orientation drawing method at a speed of 0.75 mm/min was carried out on a Z010 testing machine (Zwick/Roell, Ulm, Germany).

The contactless method consists of several stages:

(1) Setting the gap between the electroadhesive and the substrate (polymer film) to 0.1 mm;
(2) Setting the minimum peel speed to 0.000001 mm/h;
(3) Setting the specified value of electrical voltage and minimum current (10 μA);
(4) Applying electrical voltage and recording the maximum electroadhesive forces.

The study of the supramolecular structure of the polymer films was carried out by transmission electron microscopy (TEM) on EM 301 (Philips, Amsterdam, The Netherlands). The samples were prepared using the method of carbon replicas from the surface of polymer films.

## 3. Results and Discussion

Electroadhesive interactions in the field created by a constant voltage source are caused by the polarization of polymer molecules, which leads to an increase in dipole–dipole interactions between surfaces. In this regard, the molecular and supramolecular factors that significantly affect the phenomenon of electroadhesion are the polarity of the polymers (polymer films) and their degree of crystallinity (α), respectively.

**Figure 5:**
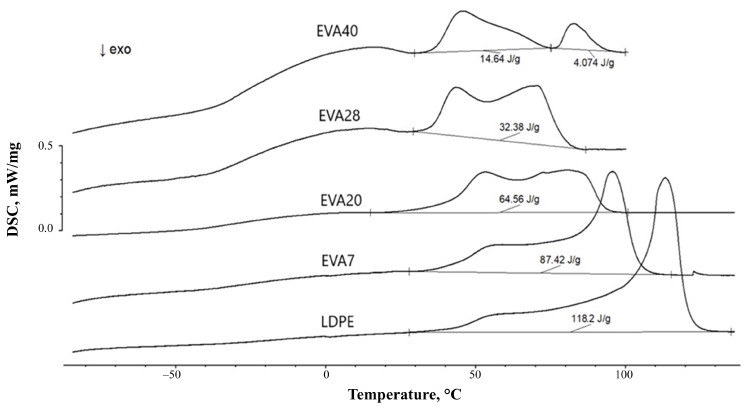
Typical DSC thermograms obtained during cooling of substrate films at a rate of 3 K/min.

To take into account the restricted mobility of macromolecular segments due to crystallinity of the studied polymer films, samples with different degrees of crystallinity were obtained. The degree of crystallinity determines the proportion of ordered regions in the polymer. Differences in the values of the degree of crystallinity for each polymer were achieved by changing the cooling rates of the pressed sample and the sample after annealing. The degree of crystallinity was calculated based on the data on the enthalpy of melting from the DSC thermograms, typical of which are shown in Figure 5. Note the fact of a decrease in the degree of crystallinity for all the studied polymer films after increasing the cooling rate of the samples heated above the melting temperature of the homopolymer or copolymer (Table 2).

The samples obtained under various conditions were subjected to electroadhesive studies at voltages of 3–8 kV and studies of free surface energy in the initial state, with the application of a voltage of 4 kV and 10 s after its removal (Table 3). Surface energy measurements were carried out at T = 22 °C and 47% humidity. The relative measurement error was 5%. For each test, at least 5 samples were analyzed. In cases where the spread of values was greater than in other systems, 7 samples were analyzed.

**Figure 6:**
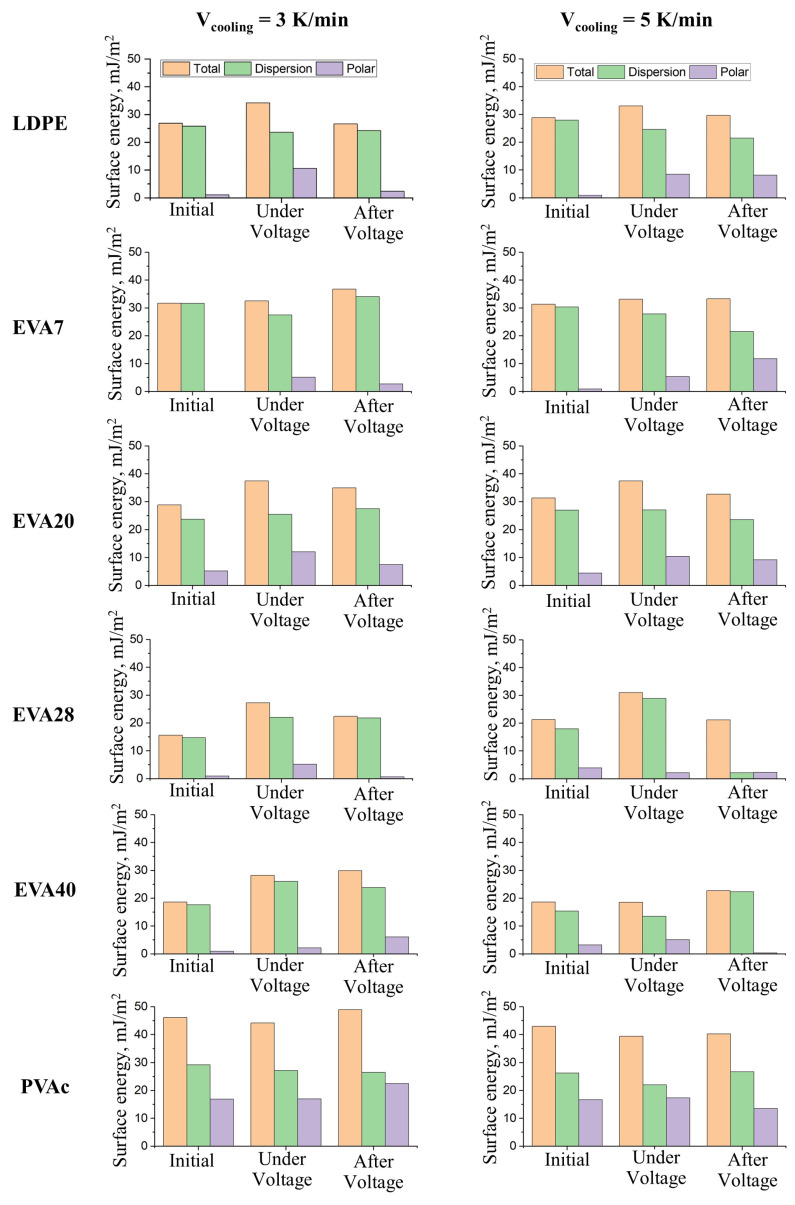
Change in surface energy and its components under the influence of an electric field (U = 4 kV) and 10 s after its removal on films with different degrees of crystallinity: LDPE, EVA7, EVA20, EVA28, EVA40 and PVAc.

Based on the obtained data (Table 3), histograms of changes in surface energy and its components under the influence of an electric field at a voltage of 4 kV were constructed for all studied objects with different degrees of crystallinity (Figure 6).

Note that the change in the degree of crystallinity of the studied polymer films within the error limits (about 5%) does not affect their surface energy characteristics. Under voltage, there is a significant increase (2 or more times) in the polar component of the surface energy, which is accompanied by some increase in the value of the total free surface energy. The exception is the PVAc film samples, where the polar component of the original sample already has high values (more than 15 mJ/m^2^) and does not change when applying electrical voltage. This behavior of the studied objects under the influence of an electric field is explained by the orientation of dipoles in the near-surface layers.

The results clearly demonstrate that 10 s after the removal of the electric voltage, the surface energy can both increase and decrease, mainly due to the polar component. This allows us to assume that the surface charge occurs due to orientational polarization, and after the removal of the voltage, dipole misorientation, leading to a change in the conformation of macromolecules, as a result of which polar groups can either come out to the surface of the sample or, conversely, turn inside the material.

**Figure 7:**
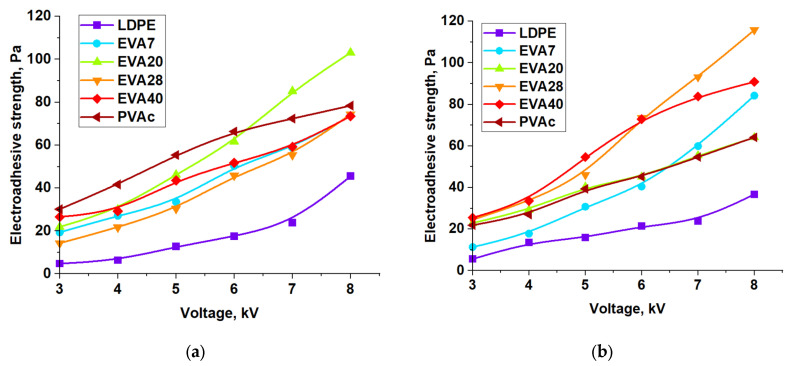
Dependence of electroadhesive strength on electrical voltage for films V_cooling_ = 3 K/min (**a**) and V_cooling_ = 5 K/min (**b**).

The obtained regularities of the influence of the conditions of obtaining samples and the electric field on the energy characteristics of the surface were tested by direct electroadhesive studies using a contactless method. The normal electroadhesive force was determined using a contactless method on film samples after 100 s of charging. The charging time is due to the need for the electroadhesive system to reach equilibrium using the contactless method, i.e., in the absence of tight contact. Figure 7 shows the dependences of the electroadhesive strength, calculated as the ratio of the normal electroadhesive force to the substrate area (polymer film), on the voltage for all the studied samples. (Here and below, the electroadhesive forces were measured at T = 295 K and 47% humidity). In the study, the applied voltage was limited to 8 kV, since at the chosen electrode fill thickness and higher voltages, dielectric breakdown occurred.

It is evident that with the growth of the electric voltage, an increase in the strength of the electroadhesive contact is observed, which is consistent with the results in [5,11,13,16]. The range of electroadhesive strength for all samples, regardless of the degree of crystallinity, is similar and is within 5–30 Pa at a voltage of 3 kV and 40–120 Pa at 8 kV. It is important to note that, regardless of the cooling rate, polyethylene films showed the lowest electroadhesive strength, which is due to their high degree of crystallinity.

Thus, when the polymer is in a highly elastic state, it retains the mobility of the amorphous phase, and the rigid confinement of macromolecules within crystalline domains hinders their polarization under an electric field. In this case, crystalline regions are characterized by lower values of dielectric constant compared to amorphous ones, which also explains the decrease in electroadhesive effects with an increase in the degree of crystallinity.

Another important factor explaining the lower electroadhesive effects in systems with polyethylene substrates is the absence of polar groups in the material. Polar functional groups in polymers create local dipole moments that affect the interaction of the polymer with other materials and with external fields. Dipole interactions occur between molecules or their parts that have a constant (occurring between polar groups in polymers) or induced (under the influence of an external electric field) moment.

The presence of acetate groups in ethylene and vinyl acetate copolymers leads to an increase in electroadhesive interactions several times compared to polyethylene. For EVA with a vinyl acetate content of 7 to 40%, this increase is from 2 to 4 times, up to 120 Pa at a constant voltage of 8 kV.

**Figure 8:**
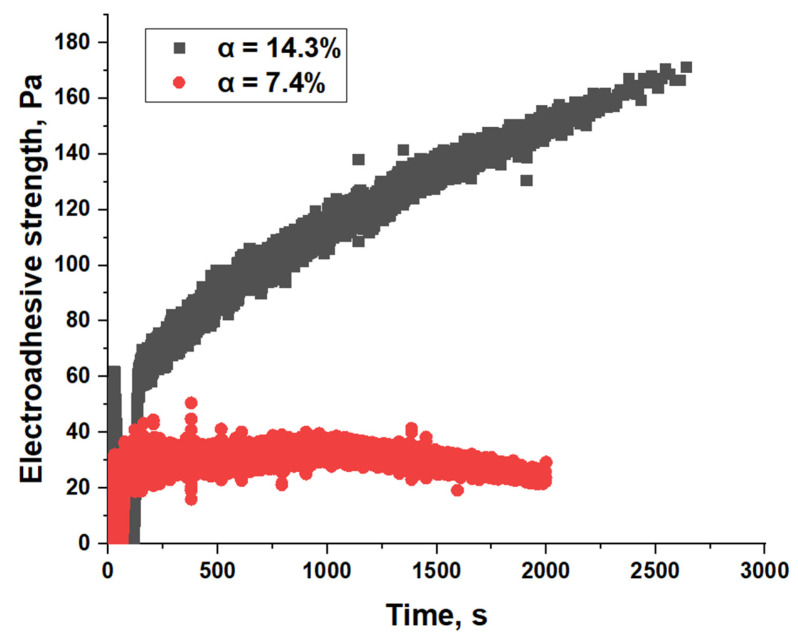
Kinetics of change in electroadhesive strength at a voltage of 5 kV in a system with EVA40 with a degree of crystallinity of 7.4 (red) and 14.3% (black).

Despite the similar range of values of electroadhesive strength for films of one copolymer, differing in crystallization kinetics and, as a consequence, characterized by different degrees of crystallinity, the effect of electroadhesion during long-term charging appears different. Using the example of the EVA40 substrate, characterized by a 2-fold higher degree of crystallinity at a lower crystallization rate (Table 2), the influence of dipole mobility under electric field conditions is shown (Figure 8). At a voltage of 5 kV, after 100 s of charging, the emerging electroadhesive forces for the substrate with a lower degree of crystallinity (α = 7.4%) reach constant values. The EVA40 sample, characterized by α = 14.3%, behaves differently in the electroadhesive system. The electroadhesive strength increases throughout the experiment and after 45 min takes a value 4 times higher compared to the sample with α = 7.4%, but does not reach equilibrium. Amorphous PVAc films do not have this effect.

The observed behavior is likely due to the slow dipole orientation process in the crystalline regions under an electric field.

**Figure 9:**
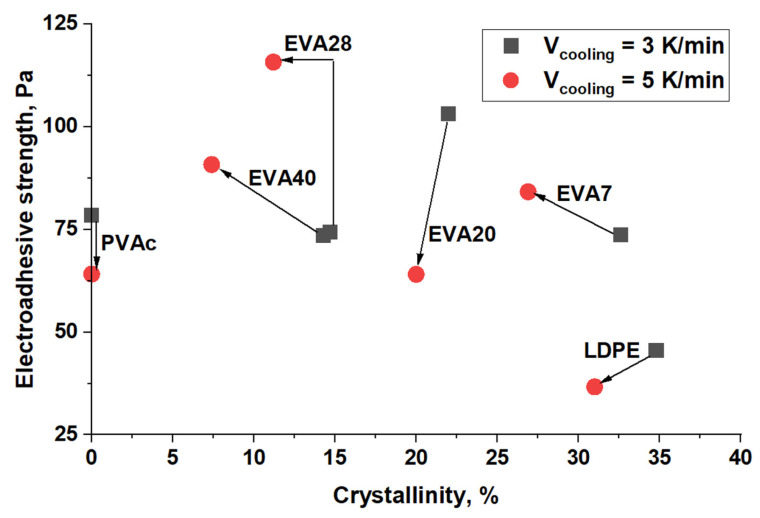
Effect of the degree of crystallinity on the electroadhesive strength at a voltage of 8 kV (from left to right—from PVAc to LDPE).

Figure 9 presents the generalized data on the influence of EVA, characterized by different values of the degree of crystallinity, on the electroadhesive strength after 100 s of charging under a voltage of 8 kV. It is evident that with such a short-term effect of the electric field on the adhesive system, despite the fact that the annealed amorphous-crystalline samples are characterized by a lower degree of crystallinity, the changes in the electroadhesive interactions are close to chaotic. The differences, as shown in Figure 8, appear at longer charging times of the adhesive system.

As noted above, in addition to the phase nature of the substrate, the magnitude of the surface energy should also influence the electroadhesive strength of the joint. With an increase in surface energy, its dispersion and polar components, the strength of the electroadhesive contact also increases. However, based on the obtained data, the value of the polar component of the surface energy is predominant.

**Figure 10:**
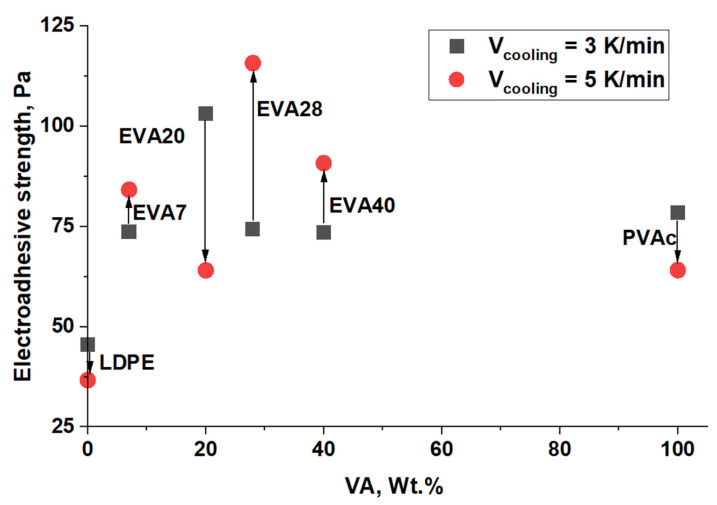
Effect of vinyl acetate content in films on electroadhesive strength at a voltage of 8 kV.

Similarly, a study was carried out to investigate the effect of vinyl acetate content (i.e., concentration of polar groups) on electroadhesive strength (Figure 10).

Regardless of the degree of crystallinity of LDPE (34% and 31%) cooled at different rates, the values of electroadhesive strength are close and amount to 45 Pa and 36 Pa, respectively. This is due to the stable supramolecular structure of LDPE films, which is slightly affected by post-processing. The obtained values of electroadhesive interactions are in good agreement with the values of the initial surface energy, where the polar component of the non-polar thermoplastic has extremely low values.

For EVA7 with a small content of vinyl acetate (polar) groups the values of electroadhesive strength increase approximately 2 times. Note that for EVA7 samples crystallized under different conditions, the values of electroadhesive strength are close (for α = 32.6% it is 84 Pa and for α = 26.9% it is 73 Pa). The obtained strength characteristics also correlate with the increase in the values of the surface energy of EVA7 films under voltage.

A further increase in the content of vinyl acetate groups does not lead to a significant increase in electroadhesive interactions. Of fundamental importance is the change in the contributions of the physical and chemical characteristics of the material to the formation of electroadhesive forces. Thus, with an increase in the content of vinyl acetate groups in EVA, the proportion of macromolecular fragments capable of polarization increases. It is important that this reduces the degree of crystallinity, which, as shown above, is one of the factors that positively affects the growth of the electroadhesive strength of joints under prolonged exposure to an electric field. Thus, we observe the influence of competing factors on electroadhesive forces.

**Figure 11:**
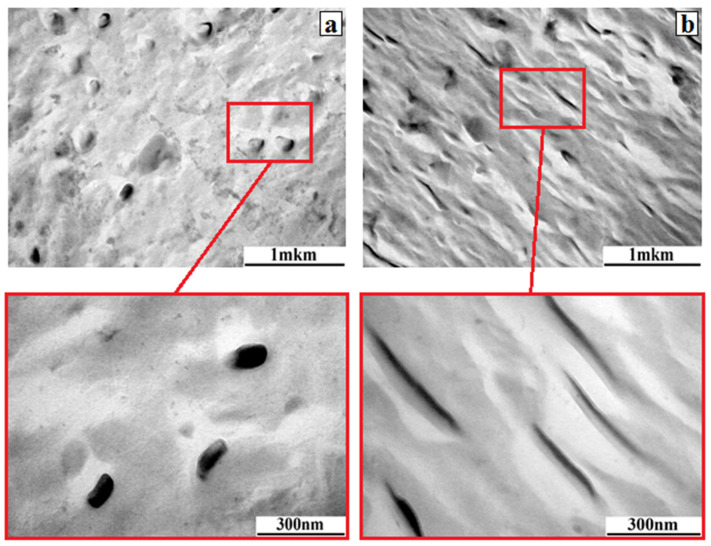
TEM images of the initial sample of EVA28 with α = 11% (**a**) and oriented with α = 15% (**b**). The figures below show characteristic enlarged areas illustrating changes in the phase structure of the samples as a result of orientation processes.

Additionally, a direct experiment was carried out to confirm the effect of the supramolecular structure of amorphous-crystalline films on electroadhesive interactions. We compared the electroadhesive interactions generated in films whose degree of crystallinity was varied not due to the kinetics of conformational rearrangements upon cooling, as shown above, but due to orientational drawing in a highly elastic state. For this purpose, two samples (80 × 40 mm^2^) were cut from one pressed EVA28 film with a thickness of 300 μm. One sample was examined in the initial state—the degree of crystallinity according to DSC data was 11%. The second sample was subjected to orientational drawing in a test machine at a speed of 0.75 mm/min. As a result of drawing, the relative elongation of the sample was 420%. It is evident that as a result of sample drawing, orientational processes lead to changes in the supramolecular structure of EVA28 (Figure 11), associated with the crystallization of ordered macromolecular chains and an increase in the degree of crystallinity to 15%.

The TEM image illustrates the effect of orientational drawing on the formation of crystalline structures extended in the direction of tensile stress. It is evident that the crystalline phase in the initial EVA28 is spherical. Applying tensile stress results in the ordering of macromolecular chains in the direction of drawing, resulting in the formation of extended crystalline structures reaching 600 nm in length and leading to a 4% increase in crystallinity.

**Figure 12:**
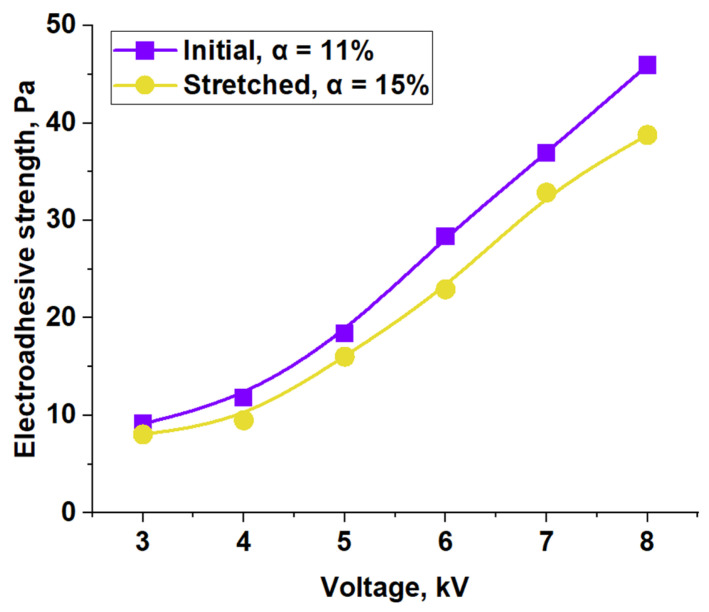
Electroadhesive strength in the system for the initial and oriented films of EVA28.

The contactless method was used to determine the forces of electroadhesive interaction along the normal after 100 s of two adhesive pairs being under voltages from 3 to 8 kV. The obtained data are presented in Figure 12 as a dependence of the electroadhesive strength of the joint on voltage. Note that the error in the obtained values did not exceed 5%, which corresponds to the confidence interval limited by the size of the marker point.

As shown earlier, with an increase in the value of constant voltage, the electroadhesive strength of the studied system increases. The influence of the degree of crystallinity before and after the orientation processes on electroadhesive interactions was established. Our finding indicates that even a slight increase in the degree of orientation of the macromolecular structure, causing an increase in the degree of crystallinity of the copolymer by 4%, leads to a decrease in the value of electroadhesive strength. This is explained by a decrease in the mobility of molecular dipoles. As a result, the orientation of dipoles under the action of an external electric field is hindered, which worsens electroadhesive interactions, and, as a consequence, reduces the value of electroadhesive strength. With an increase in constant voltage, the difference in the absolute values of the electroadhesive strength of the joints increases.

Thus, it has been established that an increase in the degree of crystallinity due to an increase in the long-range order of the supramolecular structure by means of orientational drawing of macromolecules leads to a decrease in electroadhesive interactions. While an increase in the degree of crystallinity by means of a decrease in the crystallization rate leads to the creation of an equilibrium structure with a long-range order, which is accompanied by an increase in the electroadhesive strength of the joints with a given substrate.

Summarizing the obtained results, it was found that the value of the electroadhesive strength of the joint is affected by both the content of vinyl acetate (polar groups) in the substrate film and the degree of its crystallinity. It should be noted that an increase in the number of polar groups of vinyl acetate in the material has a positive effect on the value of the electroadhesive strength, while an increase in the degree of crystallinity by increasing the equilibrium of the phase structure makes a positive contribution, and due to orientational drawing–a negative contribution to the electroadhesive strength.

**Figure 13:**
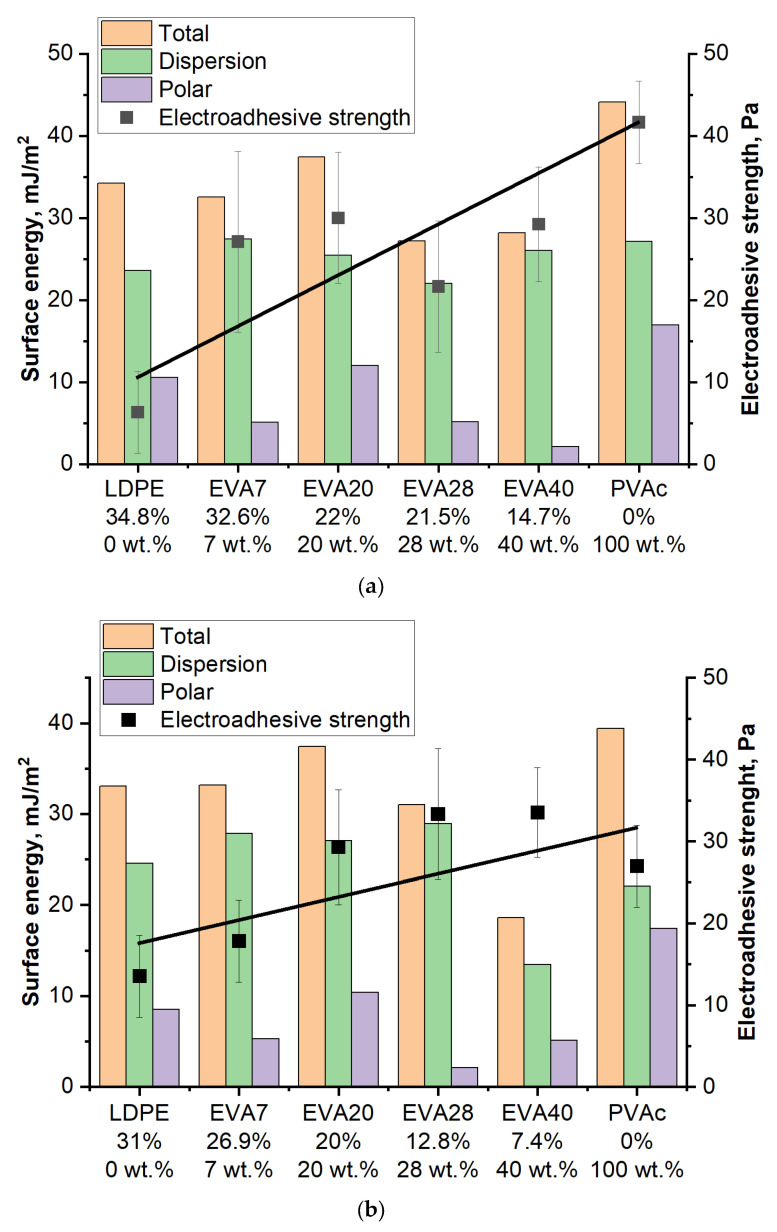
Generalized electroadhesive diagrams at a voltage of 4 kV for films with V_cooling_ = 3 K/min (**a**) and for films with V_cooling_ = 5 K/min (**b**). Explanations in the text.

Figure 13 shows generalized diagrams of surface energy and electroadhesive strength depending on the vinyl acetate content in the material and the degree of crystallinity.

In the case of films with both low and high crystallization rates (Figure 13), the electroadhesive strength at a voltage of 4 kV increases with an increase in the concentration of vinyl acetate in the material and a decrease in the degree of its crystallinity. A correlation is also observed between the value of the surface energy and the value of the electroadhesive strength. The LDPE sample is an exception. Its low electroadhesive strength is primarily due to the low polar component of the surface energy, caused by the zero concentration of vinyl acetate. With an increase in the concentration of polar groups and a decrease in the degree of crystallinity, an increase in the electroadhesive strength of the systems is observed.

## 4. Conclusions

This work presents a study of the influence of surface characteristics of substrates and the degree of crystallinity of materials on electroadhesive interactions and their changes under the influence of a constant electric field.

The effect of the voltage of the constant electric field and the phase composition of the materials on electroadhesive interactions, the surface energy of the substrate and its components has been established. The obtained data show a positive effect on electroadhesive interactions of polar groups of macromolecular chains of polymers, which is due to the improvement of their polarizability. An increase in the order of macromolecular chains when the polymer system tends to an equilibrium state also causes an increase in electroadhesive strength, while orientation processes, which also increase the degree of crystallinity, do not contribute to the growth of electroadhesive interactions, probably due to the fixation of the supramolecular structure in a nonequilibrium state. For almost all samples, a 4% decrease in crystallinity leads to an increase in electroadhesive strength of up to 1.5 times. Increasing the polar component of the substrate’s surface energy leads to a fourfold increase in electroadhesive strength. Thus, by varying the degree of crystallinity and surface energy, electroadhesive strengths of 120 Pa were achieved.

The obtained results are useful for understanding the effect of electroadhesion in polymer-polymer systems, as well as for studying the main laws of this phenomenon. For a deeper understanding of the fundamental principles of the influence of the electric field on polymer systems, it is planned to expand the scope of research from electroadhesion interactions to structure formation in polymer multicomponent systems.

Thus, this study contributes to the development of the fundamental direction of adhesive interactions in polymer systems under electric field conditions, and opens up applied prospects for the creation of functional adhesive materials with specified properties.

The main limitation of this study was dielectric breakdown, which occurred when voltage exceeded 8 kV. Future research is planned to focus on the influence of the phase structure of heterogeneous polymer systems on electroadhesive interactions. In particular, the influence of the phase structure type and phase composition of cured polymer-oligomer systems with phase decomposition on the electroadhesive effect is important to study.

## Figures and Tables

**Table 1:** Physicochemical Properties of the Initial Components.

Material	Pressing Temperature (T_P_), °C	Pressure (P), MPa	Pressing Time (t_P_), s	Cooling Rate After Pressing (V_P_), K/min	Annealing Temperature (T_A_), °C	Annealing Time (t_A_), min	Cooling Rate After Annealing (V_A_), K/min
LDPE	160	10	5	3	150	60	5
EVA7	120	10	5	3	110	60	5
EVA20	110	10	5	3	100	60	5
EVA28	95	10	5	3	85	60	5
EVA40	85	10	5	3	75	60	5
PVAc	85	10	5	3	75	60	5

**Table 2:** Degree of crystallinity of the studied objects after cooling them at different rates.

Material	α, % (V_cooling_ = 3 K/min)	α, % (V_cooling_ = 5 K/min)
LDPE	34	31
EVA7	32.6	26.9
EVA20	22	20
EVA28	14.7	11.2
EVA40	14.3	7.4
PVAc	0	0

**Table 3:** Electroadhesion strength (ES) and surface energy (γ_total_) with polar (γ^P^) and dispersion (γ^D^) component in the initial state, under voltage and after its removal.

Material		ES, Pa (4 kV)	ES, Pa (8 kV)	Surface Energy, mJ/m^2^ (4 kV)								
				Initial State			Under Voltage			After Voltage		
				γ_total_	γ^D^	γ^P^	γ_total_	γ^D^	γ^P^	γ_total_	γ^D^	γ^P^
LDPE	Without annealing	6.34	45.5	26.89	25.79	1.1	34.23	23.62	10.61	26.69	24.27	2.42
	Annealed	13.51	36.69	28.81	27.92	0.88	33.08	24.57	8.51	29.69	21.53	8.16
EVA7	Without annealing	27.11	73.6	31.69	31.65	0.04	32.55	27.45	5.1	36.78	34.06	2.72
	Annealed	17.81	84.21	31.29	30.39	0.89	33.17	27.86	5.31	33.32	21.53	11.79
EVA20	Without annealing	30.01	103.1	28.83	23.67	5.15	37.48	25.47	12.01	34.96	27.49	7.46
	Annealed	29.27	64.08	31.35	26.93	4.42	37.43	27.03	10.4	32.67	23.52	9.15
EVA28	Without annealing	30.01	74.34	15.6	14.68	0.92	27.2	22.05	5.19	22.43	21.77	0.66
	Annealed	33.33	115.8	21.86	17.94	3.92	31.04	28.93	2.11	21.17	18.85	2.32
EVA40	Without annealing	29.25	73.46	18.66	17.69	0.97	28.23	26.06	2.17	29.91	23.83	6.08
	Annealed	33.52	90.81	18.63	15.41	3.21	18.6	13.46	5.14	22.69	22.33	0.36
PVAc	Without annealing	41.66	78.39	46.15	29.24	16.91	44.13	27.14	16.99	48.96	26.49	22.47
	Annealed	26.96	64.14	42.94	26.24	16.69	39.43	22.04	17.39	40.22	26.75	13.47

## Data Availability

The original contributions presented in this study are included in the article. Further inquiries can be directed to the corresponding author.

## Abbreviations

DC source	Direct current source
LDPE	Low-density polyethylene
EVA	Ethylene-vinyl acetate copolymer
PVAc	Polyvinyl acetate
T_P_	Pressing temperature
P	Pressure
t_P_	Pressing time
V_p_	Cooling rate after pressing
T_A_	Annealing temperature
t_A_	Annealing time
V_A_	Cooling rate after annealing
DSC	Differential scanning calorimetry
TEM	Transmission electron microscopy
α	Degree of crystallinity
ES	Electroadhesion strength
γ_total_	Total surface energy
γ^D^	Dispersion component of surface energy
γ^P^	Polar component of surface energy
V_cooling_	Cooling rate
